# Artificial Intelligence Applied to the Brain-Gut Axis in Irritable Bowel Syndrome: Advancing Toward Clinical Translation

**DOI:** 10.7759/cureus.109142

**Published:** 2026-05-18

**Authors:** Temebi W Andafa, Emmanuel C Imoh, Sheriffdeen A Adekanmbi

**Affiliations:** 1 General Adult Psychiatry, Greater Manchester Mental Health NHS Foundation Trust, Manchester, GBR; 2 Psychiatry, Cygnet Hospital, Bury, GBR

**Keywords:** artificial intelligence, brain-gut axis, clinical outcome prediction, deep learning, irritable bowel syndrome, machine learning, microbiome, precision medicine

## Abstract

Irritable bowel syndrome (IBS) is one of the most common functional gut disorders affecting the global population, characterized by chronic abdominal pain and altered bowel habits in the absence of structural disease. The brain-gut-microbiota axis, a bidirectional network integrating central nervous system processing, enteric and autonomic function, immune signaling, and gut microbial ecology, provides a mechanistic framework that helps explain the substantial symptom heterogeneity and variable treatment response observed across patients. Artificial intelligence (AI) and machine learning (ML) approaches offer the ability to model complex, nonlinear relationships across high-dimensional biological datasets generated from this axis, including microbiome composition profiles, resting-state functional MRI connectivity matrices, multiomics data layers, and psychological and clinical feature sets. This narrative review evaluated primary human studies applying AI and ML to brain-gut axis data in IBS, identified through structured searches of PubMed/MEDLINE and Scopus supplemented by citation chaining, with literature included up to April 2026. Across microbiome profiling, neuroimaging, multiomics integration, and psychological feature modeling, ML approaches have demonstrated proof-of-concept performance for IBS classification and, in a smaller number of studies, for prediction of clinically meaningful outcomes, including cognitive behavioral therapy (CBT) response. A notable early signal is the integration of baseline microbiome and brain features to predict CBT response, with high reported discrimination, although these results are derived from small, single-center cohorts with only internal validation and should be regarded as hypothesis-generating. The current evidence base is limited by small single-center cohorts, reliance on internal validation, healthy-control comparators, limited external replication, and substantial overfitting and data-leakage risk in high-dimensional small-sample settings. AI and ML applications in IBS are promising but remain exploratory and are not yet suitable for routine clinical use. Clinical translation will require larger multicenter datasets, harmonized preprocessing pipelines, external validation, calibration reporting, and evaluation against clinically realistic comparators and decision points.

## Introduction and background

Irritable bowel syndrome (IBS) affects an estimated 10%-15% of the global population and represents one of the most common reasons for gastroenterology referral [1,2]. Defined clinically by chronic abdominal pain and altered bowel habits in the absence of structural disease, IBS carries a substantial burden: patients experience impaired quality of life, high rates of psychological comorbidity, and repeated healthcare contacts before a stable diagnosis is established [1,3]. Despite decades of research, no single pathophysiological mechanism explains the full syndrome, and treatment response remains highly variable and largely unpredictable at the individual level [2,4].

Contemporary IBS research is organized around the brain-gut-microbiota axis, a bidirectional network linking central nervous system (CNS) processing of visceral signals, autonomic and enteric nervous system function, immune and endocrine stress pathways, and gut microbial ecology [3,5]. This framework helps account for the heterogeneity of IBS: variability in pain sensitivity, motility, psychological comorbidity, mucosal immune activation, and microbiome composition across individuals can be positioned along the axis, and interactions among these domains may drive distinct clinical subtypes [3,5,6]. Crucially, each of these domains generates data that can, in principle, be measured and modeled.

This is where artificial intelligence (AI) and machine learning (ML) become particularly relevant. The clinical gaps AI is being asked to address are specific: current symptom-based criteria (Rome IV) classify IBS into subtypes by predominant bowel habit but do not identify biologically coherent subgroups, predict symptom trajectory, or guide selection of therapy [4,7]. Patients with identical symptom profiles may have entirely different underlying biology and respond differently to low fermentable oligosaccharides, disaccharides, monosaccharides, and polyols diet, cognitive behavioral therapy (CBT), antispasmodics, or microbiome-targeted interventions [4,6]. No validated biomarker exists that reliably predicts who will respond to which treatment. ML approaches are well-suited to this problem because they can model nonlinear relationships in high-dimensional feature spaces, including microbiome composition profiles, whole-brain functional connectivity matrices, and multiomics data layers, that are difficult to analyze with conventional univariate or regression approaches [7].

The most clinically important promise, however, is not simply improved case-control discrimination but prediction of outcomes that matter: symptom trajectory, relapse risk, and treatment response [6,7]. It must be acknowledged at the outset that most published work remains exploratory, is based on small single-center cohorts, and relies predominantly on internal validation, meaning that the distance between current evidence and deployable clinical tools is substantial. This narrative review evaluates primary human studies that apply ML and AI to brain-gut axis data in IBS, with a particular emphasis on what the evidence actually supports regarding outcome prediction and where the field needs to go to close the gap between proof-of-concept performance and clinical utility.

## Review

Methods

This narrative review was informed by structured searches of PubMed/MEDLINE and Scopus, supplemented by citation chaining, and included literature published up to April 2026. Peer-reviewed English-language human studies that apply ML or AI methods to IBS patient cohorts were considered, with an emphasis on primary empirical studies reporting clinically interpretable outcomes. Narrative reviews, expert commentaries, methodological papers, and study protocols were used for contextual background only and are not included in Table 1. No pooled statistical analysis or meta-analysis was performed because the included studies differed substantially in data types, study designs, model architectures, clinical endpoints, and validation strategies. Reported performance metrics are therefore presented as individual study findings rather than directly comparable pooled evidence.

**Table 1:** Summary of primary empirical AI/ML studies included in this review AI: artificial intelligence; ML: machine learning; IBS: irritable bowel syndrome; CBT: cognitive behavioral therapy; AUC: area under the curve; AUROC: area under the receiver operating characteristic curve; CI: confidence interval; LASSO: least absolute shrinkage and selection operator; SVM: support vector machine; ST-GCN: spatiotemporal graph convolutional network; fMRI: functional magnetic resonance imaging; 16S rRNA: 16S ribosomal ribonucleic acid; rRNA: ribosomal RNA

Study	Data type	AI/ML method	Sample size (n)	Validation type	Key metrics	Key finding	Key limitation
Jacobs et al. [6]	Gut microbiome + brain functional connectivity	Random forest classifier	84	Internal validation	AUROC = 0.96	Baseline microbiome and brain features were associated with CBT response in IBS	Small sample; internal validation only; no independent replication; high overfitting/data-leakage risk in high-dimensional small-sample setting
Saeedinia et al. [10]	Psychological and quality-of-life questionnaire data	Logistic regression	284	Internal validation and 10-fold cross-validation	AUC = 0.86 (95% CI = 0.78-0.94); accuracy = 0.83 (95% CI = 0.73-0.90)	Anxiety, depression, and somatization predict quality of life and symptom burden	No external validation; healthy-control comparator
Fukui et al. [11]	16S rRNA gut microbiome sequencing	LASSO logistic regression	111	Internal cross-validation and held-out validation	Sensitivity >80%; specificity >90%	Sparse bacterial feature set identifies IBS patients from healthy controls	Single-center cohort; healthy-control comparator; no external validation
Hollister et al. [12]	Shotgun metagenomics + metabolomics	Random forest classifier	45	Internal cross-validation	AUC = 0.93	Carbohydrate metabolism and bile acid pathways distinguish pediatric IBS from healthy controls	Pediatric cohort only; small sample; no external validation; overfitting risk
Xie et al. [17]	Resting-state fMRI connectivity	SVM	176	Internal cross-validation	Accuracy = 75%; AUC = 0.7788 (95% CI = 0.69-0.87)	Whole-brain resting-state connectivity patterns classify IBS from healthy controls	Healthy-control comparator; single-site cohort; no external validation
Zhang et al. [18]	Brain functional connectivity strength (fMRI)	SVM	61	Internal validation	Accuracy = 91.9%	Functional connectivity strength differentiates IBS from healthy controls	Small single-site cohort; internal validation only; unstable accuracy estimate
Wu et al. [19]	Dynamic resting-state fMRI connectivity	ST-GCN	158	5-fold cross-validation	Accuracy = 83.51%	Spatiotemporal brain connectivity patterns classify IBS using deep learning	Single-hospital dataset; overfitting risk; limited interpretability
Lee et al. [21]	Gut microbiota + urinary metabolomics	Logistic regression	54	Internal validation	Microbiota AUC = 0.54; metabolomics AUC = 0.65; combined AUC = 0.74	Combined microbiota and urinary metabolomics improves IBS classification over either modality alone	Small sample; no external validation; hypothesis-generating findings
Haleem et al. [22]	Psychological symptom questionnaire data	Logistic regression, decision tree, SVM	84 (test set n = 17)	Held-out test set	Balanced accuracy = 0.93; recall = 1.0; precision = 0.91	Anxiety and fatigue features identify IBS patients from healthy controls	Very small test set; unstable performance estimates; healthy-control comparator

Pathophysiological basis of the brain-gut-microbiota axis in IBS

The brain-gut-microbiota axis defines the biological domains from which ML models in IBS draw their features [8,9]. At the peripheral level, the gut microbiome communicates with the CNS through several parallel routes. Short-chain fatty acid (SCFA) and secondary bile acid production modulate enteroendocrine and immune signaling, while tryptophan metabolism influences serotonergic tone, intestinal motility, and pain sensitivity. In parallel, lipopolysaccharide and other microbial products activate mucosal immune pathways that alter barrier permeability and afferent nerve signaling [3,8,9]. The enteric nervous system integrates these peripheral signals and relays them via vagal and spinal afferents to the brainstem and cortex, where interoceptive signals are processed in regions including the insula, anterior cingulate, and prefrontal cortex [5,6]. The hypothalamic-pituitary-adrenal axis and the sympathetic nervous system provide descending modulatory inputs that can amplify or suppress visceral pain signals depending on psychological state and prior sensitization [3].

In IBS, this system appears to function in a dysregulated manner. Altered microbiome composition has been associated with reduced SCFA production and increased mucosal permeability, while heightened central sensitization lowers pain thresholds and altered threat appraisal may bias interoceptive processing toward symptom amplification [3,5,6]. These abnormalities are not uniform across patients, which makes the disorder both biologically heterogeneous and computationally challenging. For ML approaches, each mechanistic domain effectively becomes a feature space: relative abundance profiles from 16S ribosomal RNA (rRNA) or shotgun metagenomic sequencing for the microbiome; resting-state functional connectivity matrices or task-based activation maps for neuroimaging; and questionnaire-derived measures capturing psychological state, symptom severity, and quality of life for the clinical domain [7,10]. Integrating these feature spaces is a major objective of multimodal and multiomics modeling. However, each modality also carries distinct technical artifacts and batch effects that can be amplified when datasets are combined across platforms or study sites.

Microbiome-based ML

Microbiome profiling is the most frequently used data source for ML in IBS. The typical pipeline begins with 16S rRNA amplicon sequencing of fecal DNA, with the choice of variable region (most commonly V3-V4 or V4) influencing downstream taxonomic resolution. Raw sequence reads are denoised using the Divisive amplicon denoising algorithm 2 (DADA2) or QIIME2 to produce amplicon sequence variants or clustered into operational taxonomic units at 97% sequence identity. Taxonomic assignment is performed against a reference database, such as Greengenes, SILVA, or NCBI, and the choice of database can alter which taxa are detected and their apparent relative abundance. The resulting feature matrix is compositional, meaning all values sum to a constant and may violate assumptions used in many standard statistical models. Addressing this requires either rarefaction (subsampling to equal depth, which discards data) or compositional transformation, such as centered log-ratio transformation. Features are then typically reduced by filtering low-prevalence taxa and applying regularized selection methods such as least absolute shrinkage and selection operator (LASSO) or elastic net, or tree-based feature importance from random forest or XGBoost models.

Across primary microbiome ML studies in IBS, reported internal performance can appear strong, but the pattern of evidence shows a consistent disconnect between within-study accuracy and cross-cohort generalizability. The two most informative single-center studies differ in data type and population but converge on the same methodological ceiling. Fukui et al. applied LASSO logistic regression to 16S amplicon data from 85 adult IBS patients and 26 healthy controls and reported sensitivity above 80% and specificity above 90% for IBS identification [11]. Hollister et al. used the methodologically richer combination of shotgun metagenomics and untargeted metabolomics in a pediatric cohort (23 IBS, 22 healthy controls) and built a random forest classifier, achieving an area under the curve (AUC) of 0.93 [12]. Read together, the two studies show that the move from 16S to shotgun-plus-metabolomics adds analytic depth but does not change the underlying evidential structure: both use small single-center samples, both compare against healthy controls rather than clinically overlapping disorders, both rely on internal validation, and both operate in high-dimensional feature spaces in which overfitting and subtle data-leakage risks during preprocessing and feature selection are well-recognized challenges in ML applied to biological datasets [7]. The cohorts also differ in age (adult Japanese vs. US pediatric), which constrains transportability in opposite directions: neither result can be assumed to generalize to the other population without independent replication [12]. The reported performance figures may therefore reflect the upper bound of performance achievable under internally validated small high-dimensional study conditions rather than real-world generalizable accuracy.

Cross-cohort fragility is the central unresolved problem in microbiome ML and is the most informative methodological signal in this literature. Models that perform well within a single dataset frequently show substantial deterioration when tested on independent cohorts, a pattern most plausibly attributable to biological and technical variability between the cohorts (sequencing platform, variable region, reference database, extraction kit, geography, diet) rather than to a genuine generalizable signal. Comparator choice further compresses apparent performance: discrimination between IBS and clinically overlapping disorders such as functional dyspepsia is consistently weaker than discrimination between IBS and healthy controls, which limits clinical utility for the differential diagnostic question that actually arises in practice, where IBS and functional dyspepsia frequently coexist. Taken together, single-center IBS-vs.-healthy classifiers with strong internal performance and multicohort classifiers with markedly weaker external performance are not contradictory; both reflect the same underlying limitation: internal validation in small high-dimensional studies may overestimate real-world performance.

The harmonization challenges underlying this fragility are layered. Beyond variable region and reference database choices, sequencing depth, bioinformatics pipeline version, and laboratory extraction kit all introduce systematic variability that is not biological in origin. Models trained on Illumina MiSeq data using the V4 region and Greengenes taxonomy may not replicate in a cohort processed on a different platform with SILVA annotation, even if the underlying biological populations are comparable. Shotgun metagenomic sequencing avoids some of these limitations but is substantially more expensive and introduces its own batch effects related to DNA library preparation and sequencing depth. Functional inference from 16S data using tools such as Phylogenetic Investigation of Communities by Reconstruction of Unobserved States 2 adds further uncertainty, as predicted gene content is an indirect proxy for measured metabolic capacity [13].

Neuroimaging-based AI classifiers

Functional magnetic resonance imaging (fMRI) provides a window into the brain side of the brain-gut axis and has been applied to IBS classification using both task-based and resting-state paradigms. In early foundational work, visceral balloon distension paradigms demonstrated differential activation patterns in IBS compared with healthy controls, supporting a central component to visceral pain processing [14]. Subsequent synthesis work identified consistent involvement of salience, emotional arousal, and sensory processing networks in chronic visceral pain conditions, including IBS [15]. The standard resting-state preprocessing pipeline includes motion correction, spatial normalization to a standard template (Montreal Neurological Institute 152, MNI152), spatial smoothing, temporal bandpass filtering, and removal of nuisance signals using independent component analysis (ICA)-based denoising or regression of motion parameters, white matter, and cerebrospinal fluid signals. These preprocessing steps are highly sensitive to pipeline implementation choices and contribute substantially to cross-study variability in reported connectivity measures [15,16]. Functional connectivity is then estimated by correlating time series from parcellated brain regions using atlas-based parcellation schemes (e.g., the Automated Anatomical Labeling atlas or the Schaefer atlas) or data-driven ICA approaches, thereby constructing a connectivity matrix that serves as the ML feature set [16,17].

Across the three primary neuroimaging ML studies, reported accuracy ranges from 75% to 91.9%, but the spread tracks sample size and validation conservatism more than methodological superiority. The largest cohort, used by Xie et al. in 88 IBS and 88 matched healthy controls, produced the lowest point estimate (accuracy 75%, AUC = 0.7788) but reported it with a confidence interval (CI = 0.69-0.87) that makes the residual uncertainty visible. The floor and the ceiling of the interval span almost 20 AUC points [17]. Zhang et al. reported 91.9% accuracy in a smaller single-site cohort using a support vector machine (SVM) on connectivity strength features [18], but accuracy in samples of that size is highly variable across data-splitting choices, and the absence of a comparable interval makes the headline number harder to evaluate against the wider-CI Xie et al. estimate. The spatiotemporal graph convolutional network applied to dynamic functional connectivity in 79 IBS and 79 healthy controls achieved 83.51% under five-fold cross-validation, trading interpretability and increased overfitting risk for an intermediate position on the headline metric; the same study reported that its interpretability module could identify altered brain regions but could not determine causal directionality or sign of effect [19]. The crosscutting observation is that the dimensions on which these studies differ, namely feature representation (static connectivity, connectivity strength, dynamic connectivity), classifier (SVM vs. deep learning), and sample size, explain the spread in reported performance, while the dimensions on which they agree, namely internal validation, healthy-control comparator, and single-site data, explain why none of the headline figures supports a translational claim. Discrimination against a clinically realistic comparator and replication in an independent cohort are prerequisites for that claim and have not been reported for any of the three studies.

Two limitations are critical for clinical translation. First, nearly all IBS neuroimaging ML models are trained on healthy controls rather than on patients with clinically overlapping presentations, such as functional dyspepsia, inflammatory bowel disease in remission, or somatic symptom disorder. A classifier performing well in IBS-vs.-healthy comparisons may perform poorly when faced with the realistic clinical question of whether a patient with chronic abdominal pain has IBS or functional dyspepsia. Second, scanner-manufacturer and acquisition-protocol heterogeneity is a substantial barrier: functional connectivity estimates differ across scanner manufacturers (Siemens, GE, Philips), field strengths (1.5T vs. 3T), repetition times, and preprocessing pipeline implementations. Demonstrating altered connectivity in IBS is scientifically interesting; demonstrating that a connectivity classifier can be deployed clinically requires evidence that the field has not yet provided.

Multiomics integration and biological subtyping

Multiomics integration aims to move beyond single-modality feature sets and symptom-based subtypes (constipation-predominant, diarrhea-predominant, and mixed) toward biologically coherent patient clusters. The general approach combines two or more data layers, including microbiome composition, metabolomics, transcriptomics, proteomics, and neuroimaging, using integration methods ranging from early fusion (concatenating feature matrices before model training) to late fusion (training modality-specific models and combining outputs) or factor analysis approaches such as Data Integration Analysis for Biomarker discovery using Latent cOmponents, which identify latent components co-varying across omics layers [13,20].

The available multiomics evidence in IBS converges on a directional claim: combining modalities improves classification over single-modality inputs. However, the two primary studies differ in cohort design, in how transparently they expose the additive contribution of each layer, and in how directly the integrated signal is measured. Lee et al., in 27 IBS patients and 27 healthy controls, integrating 16S rRNA microbiota profiling with urinary metabolomics, made the additive logic explicit by reporting each modality alone before combining them: microbiota AUC 0.54 (essentially chance), urinary metabolomics 0.65, and the combined model 0.74 [21]. Mars et al. achieved a higher headline AUC (0.82) using longitudinal multiomics integration that identified subset-specific mechanisms invisible to any single modality [20], but the longitudinal design and breadth of layers also make it harder to isolate which modality is doing the predictive work. Beyond this contrast between transparency and richness, the studies share the limitations that constrain the literature as a whole. Both rely on internal validation in samples that are small relative to the dimensionality of the integrated feature space, both compare IBS against healthy controls rather than clinically overlapping disorders, and both depend partly on functional content inferred from 16S data rather than directly measured by shotgun metagenomics, which adds a layer of computational inference to the integrated signal. Lee et al. characterized their findings as hypothesis-generating, citing sample size, the absence of external validation, and potential dietary confounding [21]; the same characterization applies to the results of Mars et al. Neither AUC supports the translational claim that multiomics fusion produces clinically usable IBS classifiers, and the gap between the two estimates is best read as a reflection of design choices rather than as a real ranking of multiomics approaches.

Key limitations specific to multiomics integration deserve emphasis. When functional content is inferred from 16S rRNA data rather than directly measured by shotgun metagenomics, the multiomics signal contains a layer of computational inference rather than direct measurement [13]. Batch correction across omics layers introduces further complexity: sample collection timing, freeze-thaw cycles, extraction kit, and laboratory protocol all introduce systematic variability that must be corrected before integration, but overcorrection can inadvertently remove true biological signal. The cost and logistical demands of collecting harmonized multiomics data at scale remain practical barriers to clinical implementation, and feasibility in routine care settings has not been demonstrated.

Psychological and clinical feature models

ML models trained on questionnaire-derived and routine clinical data represent one of the most clinically accessible applications in IBS research, because the features, including validated symptom scales, psychological inventories, and demographic variables, are already collected in many clinical settings. Instruments such as the IBS Symptom Severity Scale, the Hospital Anxiety and Depression Scale, the Patient Health Questionnaire, and disease-specific quality-of-life measures provide structured feature sets that can be processed without specialized equipment [10,22]. In practice, however, the available primary studies share the same limitations seen across the rest of the literature: small single-center cohorts, internal validation, and healthy-control comparators.

The two primary psychological-feature studies in IBS are methodologically close, since both apply logistic regression to validated psychological and symptom inventories, both compare against healthy controls, and neither uses external validation, yet they report very different headline performance. The gap is informative because it reflects sample size and validation rigor, not real model superiority. Haleem et al. reported balanced accuracy of 0.93, recall of 1.0, and precision of 0.91, but these figures were estimated on a held-out test set comprising only 17 participants, drawn from a total cohort of 49 IBS patients and 35 healthy controls [22]. A limitation the authors acknowledged is that estimates from such small samples vary widely depending on how the data are split for training and testing and are unlikely to remain stable across resamples. Saeedinia et al. applied the same model class to a substantially larger sample of psychological and quality-of-life features and reported an AUC of 0.86 (95% CI = 0.78-0.94) under 10-fold cross-validation [10]. The Saeedinia et al. figure is numerically lower than the Haleem et al. headline but estimated more conservatively, in a larger cohort, with an explicit confidence interval. Read together, the smaller, more optimistic study and the larger, more conservative study likely reflect the same underlying signal under different sample sizes and validation conditions rather than genuinely different levels of predictive performance. The shared limitations matter more than the apparent gap. Neither study used a clinically realistic comparator such as functional dyspepsia or somatic symptom disorder, and neither was externally validated, despite the fact that questionnaire-based models, unlike microbiome or neuroimaging models, face no equipment-related barrier to multisite replication. The translational implication is consistent across both studies. Psychological features carry a usable signal for IBS-vs.-healthy classification, but neither study demonstrates the harder capability of discriminating IBS from clinically overlapping disorders or predicting individual treatment response, which is what would justify clinical deployment.

External validation is essential before any questionnaire-based model is used clinically. Questionnaire distributions vary across cultural and healthcare settings, and features identified as predictive in one country or health system may not generalize across contexts. The practical appeal of these models, given their low cost, absence of specialist equipment requirements, and reliance on routine data, makes their rigorous evaluation all the more important rather than less.

Predicting treatment response and clinical outcomes

Outcome prediction is the most clinically consequential application of AI in IBS, and the most demanding from an evidential perspective. The central question, which patient will respond to which treatment, requires models that go beyond discriminating IBS from controls and instead predict a future state in an individual patient. The evidence base for this is currently small but contains important signals.

The most widely cited example is Jacobs et al., who integrated baseline gut microbiome and brain neuroimaging features to predict response to CBT in IBS patients, reporting an area under the receiver operating characteristic curve (AUROC) of approximately 0.96 for predicting treatment response [6]. This study is notable for aligning the ML endpoint with a real clinical decision (whether to offer CBT), integrating two brain-gut axis data modalities at baseline, and demonstrating bidirectional posttreatment changes in the brain-gut-microbiome axis associated with symptom improvement, providing mechanistic plausibility alongside predictive performance [6]. These features make the study an important proof of concept.

The reported AUROC of 0.96 should not, however, be interpreted at face value. When sample sizes are modest and feature spaces are high-dimensional, as is the case here, reported metrics frequently reflect optimistic estimation arising from overfitting or from subtle data leakage during preprocessing, feature selection, or cross-validation design, rather than true generalizable performance [7]. The study used internal validation, and independent replication in an external cohort has not yet been reported. Until replicated in a separate, prospectively recruited cohort with prespecified analysis, this finding is appropriately treated as a strong hypothesis rather than a validated predictive model, and certainly not as evidence that AI-guided CBT selection is clinically ready.

The distinction between predicting binary response (responder vs. nonresponder at a single time point) and predicting symptom trajectory (the continuous path of symptoms over time) is important and underexplored. Predicting symptom trajectories over time is more complex because it requires repeated measurements and methods that account for within-subject correlation and changing confounding factors. Psychological and clinical feature models also show differential predictability across outcome types: broad psychosocial endpoints are more predictable than treatment-specific effectiveness, suggesting that the hardest target, predicting that a specific patient will respond to a specific intervention, remains largely unmet [10,22].

Digital health and wearables: emerging directions

Wearable technology and digital health platforms represent a less developed evidence base in IBS than the microbiome, neuroimaging, and multiomics literature discussed earlier, where IBS-specific proof-of-concept ML studies are already available for critical evaluation. Current published work in the wearable domain is predominantly non-IBS-specific and draws on wearable sensor data from broader gastroenterology or general health monitoring contexts [23,24]. The potential relevance to IBS outcome prediction lies in the ability to capture continuous, longitudinal data outside the clinic: wearable devices can provide autonomic nervous system proxies (heart rate variability, electrodermal activity), sleep quality metrics, physical activity patterns, and, increasingly, gut-specific signals via ingestible sensors or motility-tracking wearables [23].

For ML in IBS, these data streams are attractive because symptom variability in IBS is inherently longitudinal and within-person. Flares and remissions occur over days to weeks, often in association with stress, diet, and sleep, and ecological momentary assessment through smartphone-linked symptom diaries can provide dense within-person time series that clinic-based studies cannot capture. ML methods designed for time-series data (recurrent neural networks, hidden Markov models) could, in principle, identify predictive patterns in these continuous streams. However, this remains largely aspirational in the IBS literature; primary studies specifically applying wearable or digital phenotyping data to IBS outcome prediction are sparse, and the field is not yet at a stage where clinical utility can be assessed [23,24].

Data harmonization and batch effects: a crosscutting challenge

Across all modalities considered in this review, data harmonization is the single most important barrier to producing ML models that generalize beyond the dataset on which they were trained. Each modality carries its own sources of systematic nonbiological variability, referred to as batch effects, and when modalities are combined, these batch structures compound.

In microbiome studies, the principal sources of batch effects are the choice of 16S variable region (V3, V4, or V3-V4); the sequencing platform and chemistry; the reference database and version (Greengenes 13.8 vs. SILVA 138 vs. NCBI); the bioinformatics pipeline (QIIME2 vs. mothur vs. DADA2); and the fecal DNA extraction kit, which differentially lyses Gram-positive and Gram-negative bacteria. These factors can produce substantial differences in apparent community composition between studies and likely contribute to the reduced performance observed when classifiers are tested across external datasets.

In neuroimaging, analogous batch effects arise from scanner manufacturer and model, magnetic field strength (1.5T vs. 3T), echo time, repetition time, and preprocessing pipeline choice (FSL vs. statistical parametric mapping vs. Functional Magnetic Resonance Imaging Preprocessing Pipeline). These factors influence signal-to-noise ratio, spatial resolution, and the specific nuisance signals identified and removed, producing connectivity estimates that can differ systematically across sites [17-19]. Harmonization tools such as ComBat can remove additive and multiplicative scanner effects when applied carefully, but require sufficient within-scanner sample sizes and can remove biological variance if misapplied.

In multiomics, batch effects operate at every layer: metabolomics data are sensitive to sample collection timing, freeze-thaw cycles, and mass spectrometry instrument calibration; transcriptomic data are affected by RNA integrity and library preparation kit [20,21]. ComBat-Seq addresses batch effects in count-based omics data, but these methods have limitations and require prespecification of batch variables. When microbiome and neuroimaging datasets are combined, both sources of batch variation may interact in ways that remain poorly understood. Protocol-driven prospective multicenter studies with prespecified, harmonized data collection procedures are the only credible route to models with genuine cross-site generalizability [25].

The primary empirical AI/ML studies included in this review are summarized in Table 1. Table 1 is restricted to primary empirical AI/ML studies conducted in IBS patient populations; narrative reviews, expert commentaries, methodological papers, and study protocols cited elsewhere in this manuscript are used for background or context only and are not included as primary evidence.

Challenges

Despite promising proof-of-concept findings, several methodological limitations continue to restrict the clinical translation of AI and ML models in IBS, and these limitations should be considered when interpreting any of the performance metrics reported above.

A major limitation is sample size. Most published studies are based on relatively small single-center cohorts, often with fewer than 100 to 200 participants per group. In high-dimensional settings such as microbiome profiling and functional neuroimaging, this creates a substantial risk of overfitting, where models learn dataset-specific patterns rather than biologically meaningful signals that generalize across populations. It also creates conditions in which subtle data leakage during preprocessing, feature selection, or cross-validation design can inflate apparent performance. Reported performance metrics from studies lacking independent external testing should therefore be interpreted cautiously, particularly when very high AUC, accuracy, sensitivity, or specificity values are reported from modest sample sizes [6,11,12,22].

External validation remains uncommon across modalities. Many studies rely solely on internal cross-validation, which may substantially overestimate real-world performance. In microbiome modeling, in particular, classifiers that perform well under internal cross-validation have been observed to deteriorate markedly when applied to independent external datasets, illustrating the difficulty of transporting models across populations, sequencing pipelines, and clinical settings. These observations imply that internally validated metrics in single-center studies may overstate generalizable performance by a wide margin. Demonstrating stable performance across external cohorts is, therefore, essential before clinical implementation can be considered.

Another important limitation is heterogeneity in data acquisition and preprocessing. Microbiome studies differ in sequencing platform, variable region selection, reference database, and bioinformatics workflow, while neuroimaging studies vary in scanner type, acquisition parameters, and preprocessing methods [11,17-19]. These differences introduce batch effects that may be incorrectly learned by ML models as disease-related signals. Similar challenges arise in multiomics integration, where variability across sample handling and laboratory protocols can complicate harmonization [13,20].

Comparator selection also affects the clinical relevance of reported findings. Most current models are trained to distinguish IBS from healthy controls rather than from clinically overlapping disorders such as functional dyspepsia or inflammatory bowel disease in remission. When models have been evaluated against more clinically realistic comparators such as functional dyspepsia, performance has tended to fall substantially relative to IBS-vs.-healthy classification, illustrating how classifier performance can deteriorate in clinically realistic differential-diagnostic settings. Reported IBS-vs.-healthy AUC values, however high, therefore do not translate directly into evidence of diagnostic utility in routine practice.

Interpretability remains another barrier to adoption. Deep learning models may achieve strong classification performance but often provide limited insight into which features drive predictions or whether these features reflect biologically meaningful processes [19]. Clinicians are unlikely to adopt models that cannot be interpreted within an established clinical or pathophysiological framework. Improving transparency, calibration reporting, and reproducibility will, therefore, be important for future clinical integration [6,19].

Finally, many current studies remain exploratory and retrospective. Prospective validation within real-world clinical workflows, including evaluation of whether AI-guided prediction improves patient outcomes or treatment selection, remains largely lacking [6,20,25].

Future directions

Progress toward clinically usable outcome prediction in IBS requires specific rather than generic advances. First, external validation cohorts should become a standard expectation rather than an aspirational goal. Every primary ML study should report performance on at least one independent, prospectively recruited cohort not used in any part of model development or feature selection. Multi-center consortia are the most practical route to assembling the required sample sizes [6,20,25].

Second, standardized preprocessing pipelines should be agreed upon prospectively across participating centers before data collection begins. For microbiome studies, this means a prespecified sequencing platform, variable region, reference database version, and bioinformatics pipeline. For fMRI studies, it means harmonized acquisition parameters and a single preprocessing implementation with preregistered parameter choices [17,19].

Third, calibration reporting should accompany discrimination metrics. AUC and accuracy describe the rank-ordering of predicted probabilities, not their accuracy. A model that ranks patients correctly but assigns poorly calibrated probabilities will mislead clinicians about actual risk. Calibration curves and Brier scores should be reported alongside AUC in all prediction studies [6,10].

Fourth, studies should evaluate models against clinically realistic comparators, including functional dyspepsia, inflammatory bowel disease in remission, and somatic symptom disorder, rather than exclusively healthy controls, and should report performance on the clinical decision the model is intended to support [17].

Fifth, IBS-specific digital phenotyping studies using continuous wearable and ecological momentary assessment data are needed. Within-person longitudinal prediction is likely to be more clinically actionable than cross-sectional classifiers, and is technically feasible with currently available wearable technology [23,24].

## Conclusions

AI and ML applied to the brain-gut axis show early proof-of-concept potential for improved stratification and outcome prediction in IBS, but the evidence base is early, heterogeneous, and predominantly proof-of-concept. Across microbiome, neuroimaging, multiomics, and psychological feature modalities, models demonstrate the ability to identify IBS-associated patterns, and a smaller number of studies show early feasibility for outcome prediction, most notably studies integrating baseline gut microbiome and brain neuroimaging features to predict CBT response with high reported discrimination. These findings are encouraging, but they derive from small single-center cohorts with internal validation only, are subject to substantial overfitting and data-leakage risk in high-dimensional small-sample settings, and should be treated as hypotheses requiring replication rather than as validated predictive tools.

A consistent methodological lesson emerging across all modalities is that internal validation in small single-center studies may overestimate generalizable performance. Models that perform well within their training dataset frequently show marked deterioration when tested on independent cohorts, reflecting the combined influence of small sample sizes, high-dimensional feature spaces, and uncontrolled batch effects across sequencing platforms, imaging protocols, and laboratory procedures. High AUC, accuracy, sensitivity, or specificity values reported from such designs should not be interpreted at face value or assumed to reflect reliable real-world clinical performance.

Closing the gap between proof-of-concept performance and clinical utility will require external validation as a standard expectation, harmonized multicenter data collection, calibration reporting alongside discrimination metrics, evaluation against clinically realistic comparators (such as functional dyspepsia, inflammatory bowel disease in remission, and somatic symptom disorder) rather than healthy controls, and prospective evaluation within real clinical workflows. Until these standards are met, AI and ML applications in IBS should be regarded as exploratory and not yet suitable for routine clinical use, and reported performance figures should not be used as a basis for diagnostic or therapeutic decisions in routine practice.
